# Frequency specificity of aberrant triple networks in major depressive disorder: a resting-state effective connectivity study

**DOI:** 10.3389/fnins.2023.1200029

**Published:** 2023-06-30

**Authors:** Ying Li, Linze Qian, Gang Li, Zhe Zhang

**Affiliations:** ^1^Department of Electronics and Information Engineering, Lanzhou Institute of Technology, Lanzhou, China; ^2^College of Biomedical Engineering and Instrument Science, Zhejiang University, Hangzhou, China; ^3^Key Laboratory of Urban Rail Transit Intelligent Operation and Maintenance Technology & Equipment of Zhejiang Provincial, Zhejiang Normal University, Jinhua, China; ^4^College of Mathematical Medicine, Zhejiang Normal University, Jinhua, China; ^5^School of Physics, Hangzhou Normal University, Hangzhou, China; ^6^Institute of Brain Science, Hangzhou Normal University School of Basic Medical Sciences, Hangzhou, China

**Keywords:** major depressive disorder, triple networks, frequency specificity, effective connectivity, classification

## Abstract

Major depressive disorder (MDD) has been associated with aberrant effective connectivity (EC) among the default mode network (DMN), salience network (SN), and central executive network (CEN)—collectively referred to as triple networks. However, prior research has predominantly concentrated on broad frequency bands (0.01–0.08 Hz or 0.01–0.15 Hz), ignoring the influence of distinct rhythms on triple network causal dynamics. In the present study, we aim to investigate EC alterations within the triple networks across various frequency bands in patients with MDD. Utilizing a data-driven frequency decomposition approach and a multivariate Granger causality analysis, we characterized frequency-specific EC patterns of triple networks in 49 MDD patients and 54 healthy controls. A support vector machine classifier was subsequently employed to assess the discriminative capacity of the frequency-specific EC features. Our findings revealed that, compared to controls, patients exhibited not only enhanced mean EC within the CEN in the conventional frequency band (0.01–0.08 Hz), but also decreased mean EC from the SN to the DMN in a higher frequency band (0.12–0.18 Hz), and increased mean EC from the CEN to the SN in a sub-frequency band (0.04–0.08 Hz); the latter was significantly correlated with disease severity. Moreover, optimal classification performance for distinguishing patients from controls was attained by combining EC features across all three frequency bands, with the area under the curve (AUC) value of 0.8831 and the corresponding accuracy, sensitivity, and specificity of 89.97%, 92.63%, and 87.32%, respectively. These insights into EC changes within the triple networks across multiple frequency bands offer valuable perspectives on the neurobiological basis of MDD and could aid in developing frequency-specific EC features as potential biomarkers for disease diagnosis.

## 1. Introduction

Major depressive disorder (MDD) constitutes a debilitating psychiatric affliction, impacting approximately 7% of the global population (Mathers and Loncar, 2006). The condition is typified by persistent emotions of sadness, guilt, and worthlessness, as well as heightened suicide risk (Gotlib and Joormann, 2010). Despite the unidentified neurological substrates underpinning MDD, contemporary neuroimaging investigations have revealed functional connectivity (FC) anomalies across extensive brain networks (Otte et al., 2016; Li et al., 2018; Yan et al., 2019; Peng et al., 2020; Yang et al., 2021). These findings have reconceptualized MDD as a disorder of brain network dysfunction, offering novel perspectives for understanding its pathophysiology.

The triple-network model serves as a notable approach for investigating brain dysconnectivity in psychiatric disorders, delineating a core connectivity pattern that underlies cognitive, perceptual, affective, and social functions, encompassing the default mode network (DMN), salience network (SN), and central executive network (CEN) (Menon, 2011). In MDD patients, FC disruptions within these triple networks have been recurrently observed (Balaev et al., 2018; Cheng et al., 2018; Yan et al., 2019). Notably, the bulk of prior studies gauged FC by calculating Pearson’s correlation between time series of two given brain regions, hindering exploration of the influence of one brain region over another. In contrast, more recent studies have begun to probe the effective connectivity (EC) within MDD’s triple networks, examining the causal or directed influence of one brain region upon another. For example, research employing spectral dynamic causal modeling uncovered weakened connection strength from the SN to the CEN region in MDD patients (Kandilarova et al., 2018). Another study involving 336 MDD patients revealed both increased and reduced ECs from the SN regions (e.g., temporal pole) to other brain regions (Rolls et al., 2018). These findings collectively suggest that abnormal directed influences between triple network regions may be pivotal in MDD etiology. However, these studies primarily focused on MDD-induced EC changes within a broad frequency band (0.01–0.08 Hz or 0.01–0.15 Hz), potentially obscuring information regarding physiological fluctuations at specific frequencies.

The human brain, a biologically intricate system, features myriad oscillatory waves working in concert (Buzsáki and Draguhn, 2004; Samaha et al., 2020). Blood oxygen level-dependent (BOLD) signals at distinct frequency bands can partially reflect these neural processes and corresponding physiological functions (Zuo et al., 2010; Cole and Voytek, 2017; Hu et al., 2021). Previous MDD research has often identified frequency-specific alterations in spontaneous brain activity and connectivity. For instance, a study examined resting-state signal amplitude variability across two discrete frequency bands (slow-5: 0.01–0.027 Hz and slow-4: 0.027–0.073 Hz), revealing that the balance between the DMN and sensorimotor network favored the DMN in slow-5 and correlated with clinical depression symptom scores (Martino et al., 2016). Similarly, a study analyzing FC patterns in bipolar disorder depression across slow-5 and slow-4 found increased long-range FC density in the left lingual gyrus in slow-5 and decreased density in slow-4 (Yang et al., 2021). These results suggest that analyzing functional abnormalities of MDD at multiple frequencies is more rational than examining the routine band. To date, no prior study has explored EC at various low-frequency bands in MDD patients, and the impact of different rhythms on triple network causal processes in the disorder remains uncertain.

This study endeavors to assess EC changes of the triple networks at disparate frequency bands in MDD patients. By employing a data-driven method called complete ensemble empirical mode decomposition with adaptive noise (CEEMDAN) (Colominas et al., 2014), we initially decomposed BOLD oscillations into five distinct frequency bands. Subsequently, we quantified frequency-specific EC patterns among triple network components by integrating group independent component analysis (GICA) with multivariate Granger causality analysis (mGCA). We also conducted a correlation analysis to evaluate the association between EC changes and clinical measures in patients. Moreover, we utilized a support vector machine (SVM) to ascertain whether frequency-specific EC features of the triple networks could facilitate the differentiation of MDD patients from healthy controls (HCs). Based on prior evidence indicating disrupted triple networks in MDD, we hypothesized that (a) the EC in the triple networks would exhibit alterations in patients across various frequency bands; and (b) frequency-specific EC could serve as a biomarker for distinguishing patients from controls.

## 2. Materials and methods

### 2.1. Participants

This study enrolled 58 patients with MDD and 57 age-, gender-, and education-matched HCs. MDD patients were recruited from Gansu Provincial Hospital, while the HCs were obtained through newspaper advertisements. MDD diagnosis followed the Diagnostic and Statistical Manual of Mental Disorders, Fifth Edition (DSM-V). Exclusion criteria for MDD patients encompassed acute physical illness history, substance abuse/dependence, head trauma resulting in unconsciousness, claustrophobia, bipolar depression, and other neurological disorders. Hamilton Depression Scale (HAMD) and Hamilton Anxiety Scale (HAMA) evaluated depression and anxiety severity in MDD individuals. HCs were interviewed using the DSM-IV non-patient edition. All participants provided written informed consent before study procedures. The study adhered to the Helsinki Declaration and received approval from the Ethics Committee of Gansu Provincial Hospital. After head motion exclusion, the remaining 49 MDD patients and 54 HCs were included in the subsequent analyses. Demographic and clinical characteristics of participants are displayed in Table 1.

**Table 1:** Demographics and clinical characteristics of the participants.

Characteristics	MDD (*n* = 49)	HC (*n* = 54)	*p* value
Age (years)	34.09 ± 12.06	34.56 ± 12.16	0.83^a^
Handedness (right/left)	49/0	54/0	0.99^b^
Gender (males/females)	27/22	29/25	0.87^b^
Antidepressants (yes/no)	7/42	–	–
HAMD	17.40 ± 5.89	–	–
HAMA	17.05 ± 7.36	–	–
Duration of illness (years)	6.83 ± 7.88	–	–
Mean FD	0.14 ± 0.09	0.14 ± 0.07	0.86^a^

Values represented mean ± SD. SD, standard deviation; HAMD, Hamilton depression scale; HAMA, Hamilton anxiety scale; FD, frame-wise displacement; MDD, major depressive disorder; HC, healthy control.

a *p* value was obtained by two-sample *t* tests.

b *p* value was obtained by Chi square test.

### 2.2. Data acquisition and preprocessing

Resting-state fMRI data for all participants were collected on a 3.0 T scanner (Siemens, Erlangen, Germany) using a single-shot, gradient-recalled echo planar imaging sequence. Scanning parameters were as follows: repetition time (TR) = 2000 ms, echo time (TE) = 30 ms, flip angle (FA) = 90°, slice thickness = 3.5 mm, in-plane matrix = 64 × 64, field of view (FOV) = 220 mm × 220 mm, and 33 slices covering the entire brain. Participants were instructed to remain silent and awake with eyes closed, minimize movement, and let their thoughts wander during the scan. Data preprocessing employed DPARSF software^1^ based on the SPM12 toolbox,^2^ including discarding the initial 10 functional images, realignment, time-slicing, head motion correction, spatial normalization to the Montreal Neurological Institute (MNI) template, linear detrending, and nuisance covariate regression. Participants with head movement exceeding 1.5 mm translation or 1.5° rotation or with mean frame-wise displacement (FD) values over 0.5 mm were excluded from the analysis.

### 2.3. Definition of frequency of interest

**Figure 1:**
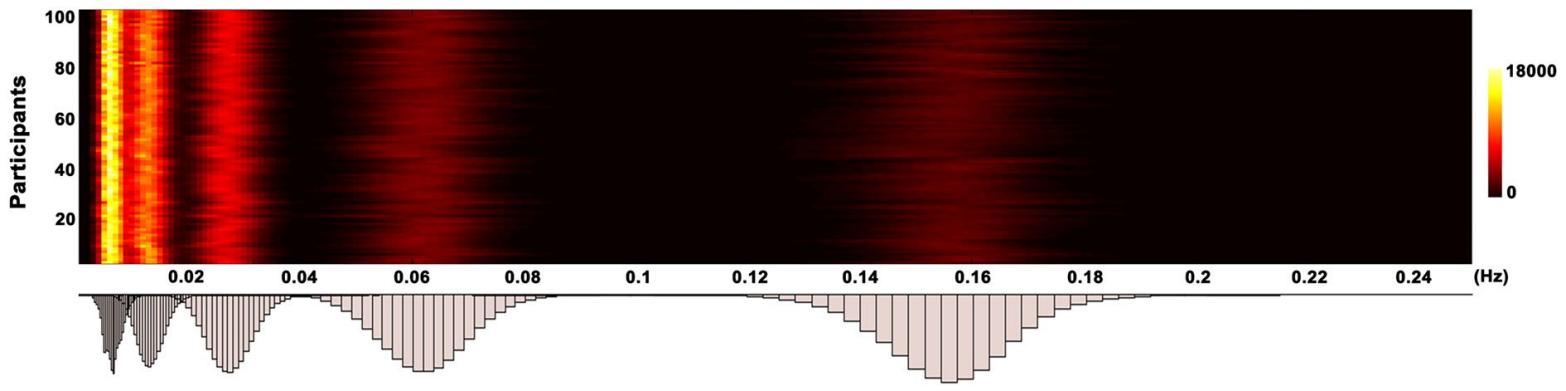
Histogram of frequency distribution. The histograms of HWF distributions show the first five intrinsic mode functions of each voxel in the whole-brain gray matter across all participants by using the CEEMDAN approach. The color bar represents the number of voxels with HWF equal to the frequency on the horizontal axis in the whole-brain gray matter. HWF, Hilbert weighted frequency; CEEMDAN, complete ensemble empirical mode decomposition with adaptive noise.

A data driven CEEMDAN method was adopted to decompose BOLD signals into distinct frequency bands without rigidly predefined band-pass filters. Briefly, a time series x(t) can be represented as $x\left(t\right)=\overset{K}{\underset{i=1}{\sum }}IMF_{i}\left(t\right)+r\left(t\right)$ , where $IMF_{i}\left(t\right),i=1,2,\cdots ,K$ is a set of intrinsic mode functions, r(t) is the monotonic residue signal, and *t*, *i*, and *K* are the length of scanning time, the order of IMF, and the number of IMF, respectively. CEEMDAN employs an iterative technique, the sifting algorithm, based on Empirical Mode Decomposition (EMD) to extract IMFs. This algorithm comprises the following steps: (1) Initially, EMD is used to derive the first residual component; (2) The first IMF is subsequently calculated by subtracting this first residual component from the original signal; (3) The second residual component is then estimated, and this defines the second IMF; (4) These steps are iteratively repeated until the final IMF is successfully extracted. Note that each IMF component occupies a distinct frequency band. In particular, the first and last IMF occupies the highest and lowest frequency bands, while the remaining IMF occupy the frequency bands in between. After decomposition, the Hilbert weighted frequency (HWF) was utilized to represent the mean oscillation frequency of an intrinsic mode function (IMF) using amplitude and phase from the instantaneous spectrum. HWF distribution histograms for each participant were calculated by determining the HWF of each IMF. A frequency of interest (FOI) was derived from each component of the HWF distribution within 95% confidence intervals to isolate frequency bands and minimize the influence of extreme values. Detailed procedures for defining FOIs can be found in a previous study (Zhang et al., 2018). Five frequency intervals (0.12–0.18 Hz, 0.04–0.08 Hz, 0.02–0.04 Hz, 0.01–0.02 Hz, and 0–0.01 Hz) were chosen as FOIs to represent EC alterations in MDD patients (Figure 1). To simplify, these intervals were designated as FOI-1 to FOI-5, with FOI-1 representing the highest frequency interval and FOI-5 the lowest. Additionally, for comparative purposes, the conventional frequency band ranging from 0.01 to 0.08 Hz was selected as the normal frequency of interest (FOI-N).

### 2.4. Triple network identification

A spatial GICA was applied to decompose resting-state fMRI data, using the GIFT toolbox. Global signal regression was first implemented within the GICA framework, where the global mean signal per time point was removed as a standard processing step preceding PCA. PCA was employed to condense subject-specific data into 120 principal components. Subsequently, we concatenated subject-reduced data across time for all participants, reducing them into 100 ICs using the infomax algorithm (Bell and Sejnowski, 1995). To ensure decomposition reliability and stability, the infomax ICA algorithm was run 20 times using ICASSO. We employed a group information-guided ICA approach to reconstruct subject-specific spatial maps and corresponding time courses after estimating group spatial maps. ICNs among the 100 ICs were identified through a combination of spatial template-matching and visual inspection, using templates derived from ICA analyses as previously described (Allen et al., 2014; Tu et al., 2019). Components were evaluated based on the following criteria: (1) peak activation coordinates primarily located in gray matter; (2) minimal spatial overlap with known vascular, ventricular, motion, and susceptibility artifacts; (3) time courses predominantly characterized by low-frequency fluctuations (Kim et al., 2017; Fiorenzato et al., 2019). We further post-processed the time courses of ICNs to remove residual noise sources by detrending linear, quadratic, and cubic trends, regressing the six realignment parameters and their temporal derivatives, despiking detected outliers, and applying low pass filtering with a cutoff frequency of 0.15 Hz.

### 2.5. Granger causality analysis

We employed GCA to investigate EC between ICs in resting-state fMRI data, a widely-used method for predicting one system’s causal influence over another (David et al., 2008; Deshpande and Hu, 2012). GCA, unlike other EC measures, quantified causal influence among multiple brain regions in a data-driven manner, without necessitating a predefined model (Deshpande and Hu, 2012). GCA’s concept can be described as follows: for two signals *s*_1_(*t*) and *s*_2_(*t*), if knowing the past information of *s*_1_(*t*) aids in predicting *s*_2_(*t*)'s future, *s*_1_(*t*) has a causal influence on *s*_2_(*t*). In this study, we evaluated the causal influences among the time courses of DM components using the mGCA method (Liao et al., 2011). For each participant, the time courses set was defined as *S*(*t*) = (*s*_1_(*t*), *s*_2_(*t*), …, *s_n_*(*t*)), where *n* denotes the number of DM components. The influence from all other seed components to target component *k* was evaluated by the multivariate auto-regressive model as follow:


$$s_{k}\left(t\right)=\overset{p}{\underset{m=1}{\sum }}C_{k}\left(m\right)S\left(t-m\right)+R_{k}\left(t\right)$$

where *p*, *C_k_*, *S* and *R* denote the auto-regressive model order, model coefficient matrix, time courses matrix of different components and residual error matrix, respectively. The model order *p* was determined using Akaike’s information criterion and the model coefficient matrix *C_k_* was calculated using a standard least squares optimization, respectively. We further calculated random-effect Granger causality maps for each participant to evaluate the statistical significance of Granger causality results, corrected with a false discovery rate (*p* < 0.05).

### 2.6. Classification analyses

We examined whether frequency-specific EC could differentiate MDD patients from controls by employing the SVM classifier. SVM is a widely-used, high-performing supervised learning model that projects low-dimensional, non-separable data into high-dimensional, separable data (Cortes and Vapnik, 1995). A 10-fold cross-validation methodology was adopted, which incorporated nested feature selection and classifier training using a Lib-SVM framework based on a linear kernel function with parameter optimization (Pereira et al., 2009). A two-step feature selection strategy was implemented to identify the optimal feature subset and minimize the risk of overfitting. This approach comprised two components: the Minimum Redundancy and Maximum Relevance (MRMR) method and the Support Vector Machine Recursive Feature Elimination (SVM-RFE) technique. Specifically, the MRMR was employed to exclude features with weak discriminative capabilities, and the SVM-RFE was further utilized for more refined feature selection. The dataset was randomly partitioned into 10 approximately equal subsets. For each iteration, a single subset served as the test dataset, while a model induced from the remaining nine subsets was tested using a classification algorithm. Each subset was used precisely once as the testing data, this process was iterated 10 times. The feature selection was incorporated within the 10-fold cross-validation and was solely performed on the training set. Meanwhile, the chosen features were applied to the testing set. The entire procedure was conducted 10 times, and the mean value derived from the 100 results was taken as the final measure of accuracy. Additionally, we employed receiver operating characteristic (ROC) curves and the area under the curves (AUC) to evaluate EC’s potential as a marker for discriminating MDD patients from controls. The LIBSVM 3.22 Matlab toolbox facilitated all classification analyses.

### 2.7. Statistical analysis

A permutation testing (10,000) was employed to evaluate group differences in EC metrics between patients and controls. The significance level was established at a threshold of *p* < 0.05, with false discovery rate (FDR) correction. Spearman’s correlation analysis was performed to assess the relationship between EC metrics and clinical symptoms, controlling for age, gender, and mean FD. Correlations with *p* < 0.05 were considered significant, FDR-corrected.

## 3. Results

### 3.1. Independent components of the triple networks

**Figure 2:**
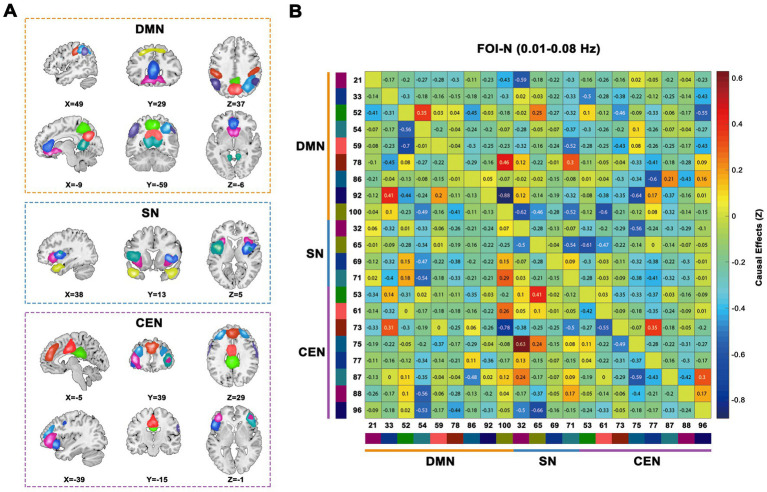
Triple-networks identified by a group ICA. **(A)** Three resting-state networks (DMN, SN, and CEN) were identified by grouping subsets of the 21 ICs. **(B)** Whole sample averaged causal influences between ICs was computed in conventional frequency band (0.01–0.08 Hz). Index numbers of ICs are written on the left and bottom side of the matrix, along with a color-coded legend, which matches to the overlaid colors of the spatial maps in **(A)**. ICA, independent component analysis; DMN, default mode network; SN, salience network; CEN, cognitive executive network; ICs, independent components.

As illustrated in Figure 2, we identified 21 ICs via group ICA and subsequently classified them into three subsets: DMN (IC 21, 33, 52, 54, 59, 78, 86, 92, and 100), SN (IC 32, 65, 69, and 71), and CEN (IC 53, 61, 73, 75, 77, 87, 88, and 96). Figure 2B present the group-averaged causal influences between each IC pair of the triple networks in the conventional low-frequency band (0.01–0.08 Hz) and the corresponding EC matrix. Detailed activation information for these ICs can be found in Supplementary Table S1.

### 3.2. Frequency-specific EC alterations in MDD

**Figure 3:**
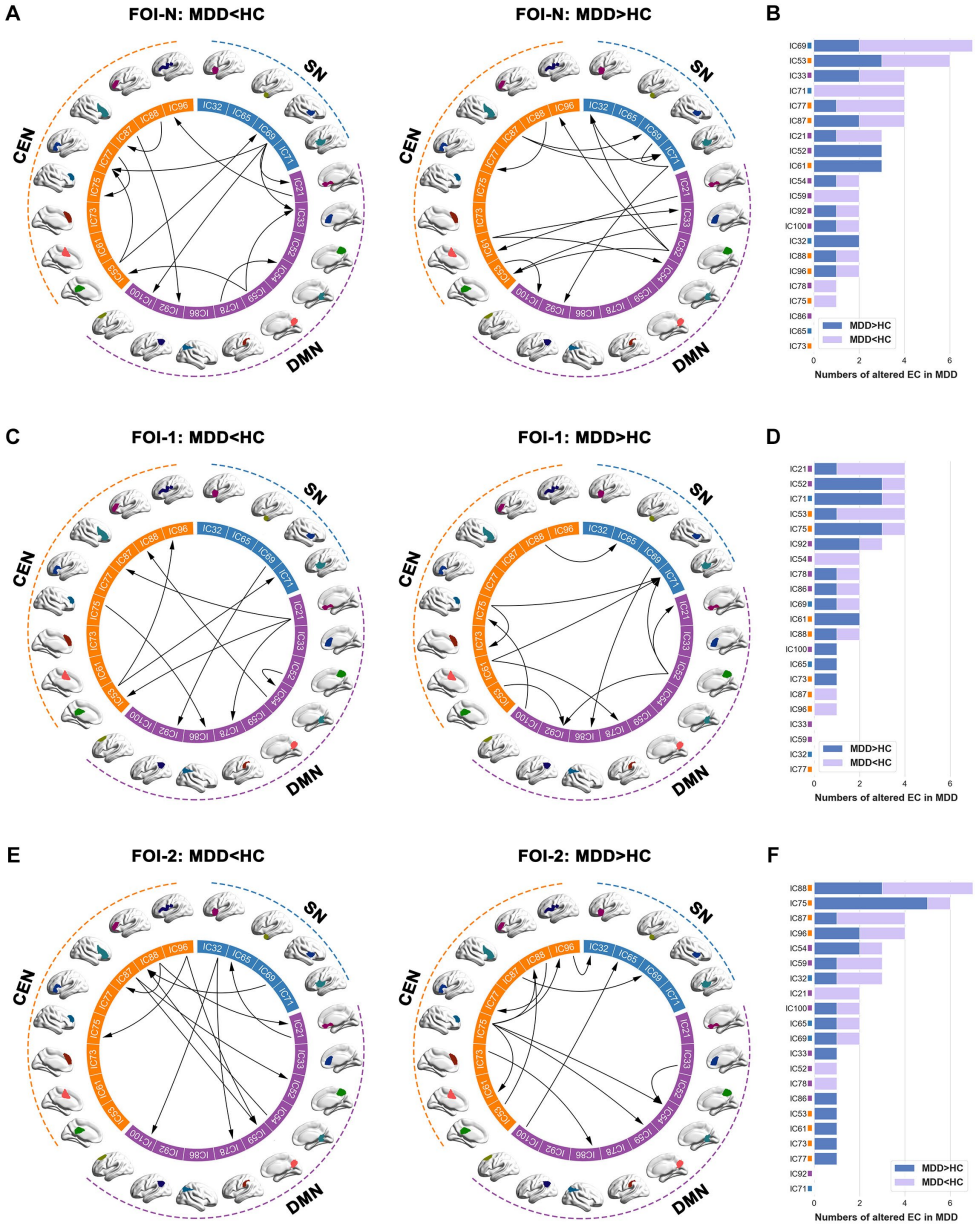
Group differences of EC across different frequency bands. **(A,C,E)** Significant between-group differences of EC in FOI-N, FOI-1 and FOI-2, and **(B,D,F)** the corresponding difference numbers of individual IC from and to the rest of ICs in each frequency band. The arrows indicate the directions of causal influences. Two sample *t*-test, significant level was set at *p* < 0.05, FDR-corrected. EC, effective connectivity; FOI, frequency of interest; IC, independent component.

We examined the EC patterns and observed significant differences between the two groups. As depicted in Figure 3, patients exhibited widespread alterations in the EC patterns of the triple networks across FOI-N, FOI-1, and FOI-2 compared to the controls (*p* < 0.05, FDR-corrected). Notably, IC69 (insula), IC21 (medial frontal gyrus), and IC88 (inferior frontal gyrus) demonstrated the most EC differences compared to other RNSs in FOI-N, FOI-1, and FOI-2, respectively. The corresponding statistical results within each FOI are provided in Figures 3B,D,F. No significant differences between groups were observed in other frequency bands.

**Figure 4:**
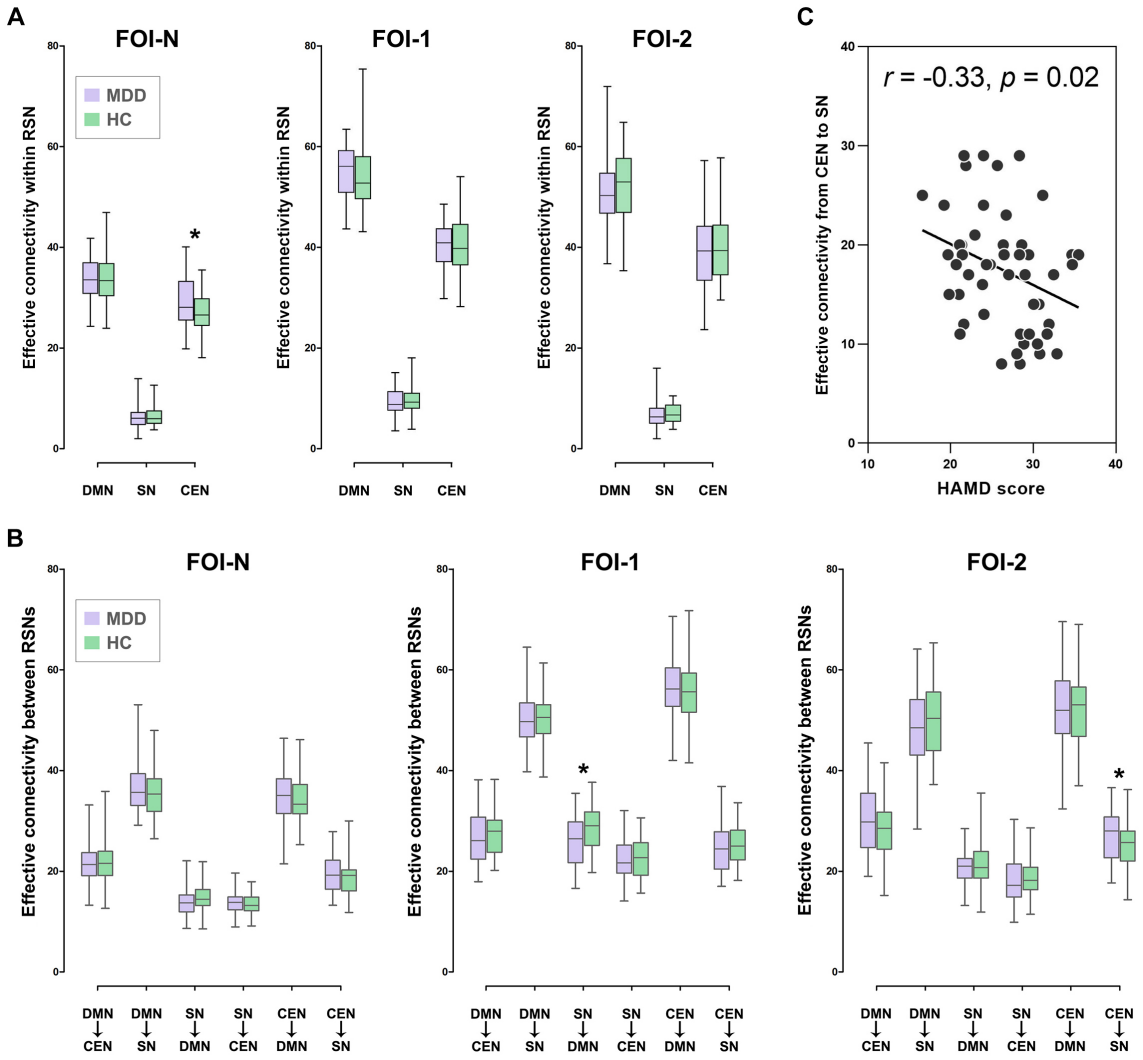
Group differences of EC in RSNs over frequency bands. **(A)** Group differences of EC within RSN in FOI-N, FOI-1 and FOI-2. **(B)** Group differences of EC between RSNs in FOI-N, FOI-1 and FOI-2. **(C)** Correlation between the HAMD score of patients and EC with significant group differences. Note that the values presented are the average ECs of all ROIs in each RSN. *p < 0.05, FDR-corrected. HAMD, Hamilton depression scale.

We then explored the causal influences within and between subsets of the triple networks across frequency bands. We discovered that the mean EC within the CEN was significantly increased in patients compared to controls in FOI-N (*p* = 0.016, FDR-corrected; Figure 4A). Meanwhile, the mean EC from SN to DMN and from CEN to SN were significantly decreased in patients compared to controls in FOI-1 (*p* = 0.011, FDR-corrected) and FOI-2 (*p* = 0.014, FDR-corrected), respectively (Figure 4B). These analyses suggest that the causal influences of the triple networks are altered in patients with MDD in a frequency-specific manner.

We further investigated whether EC metrics with significant group differences correlated with patients’ clinical symptoms and found that the mean EC from CEN to SN in FOI-2 was negatively correlated with HAMD scores in patients (*r* = −0.33, *p* = 0.02, FDR-corrected; Figure 4C). This indicates that lower causal influences from CEN to SN in FOI-2 are associated with greater disease severity.

### 3.3. Classification performance

**Figure 5:**
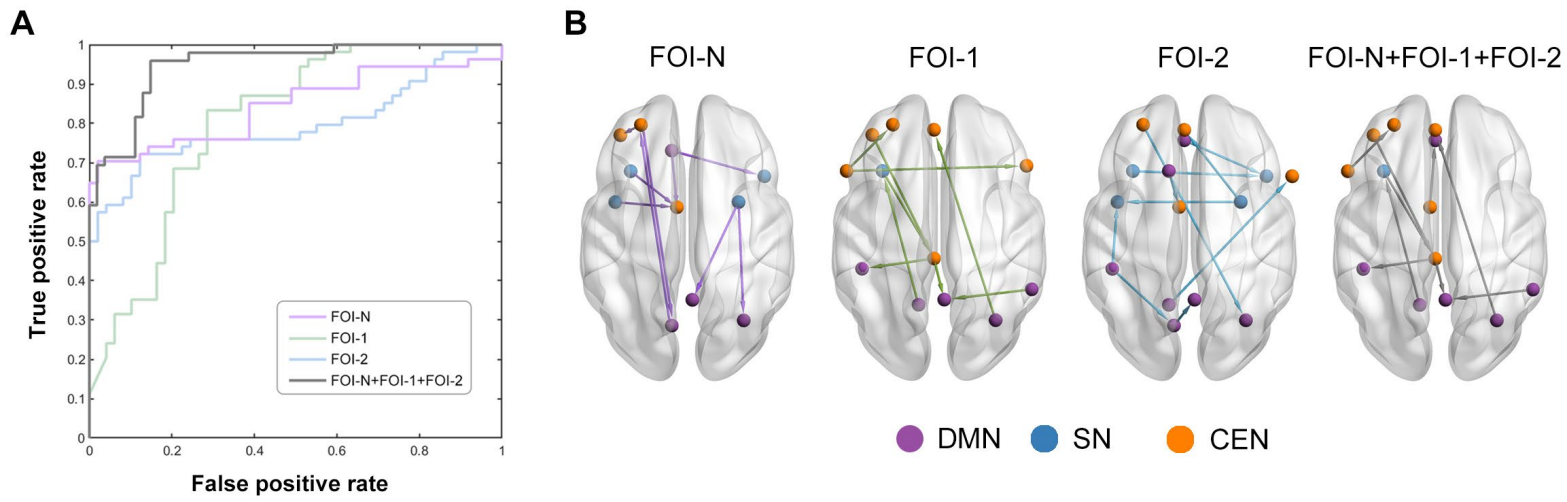
Classification performance by using EC features. **(A)** The average receiver operating characteristic curves of classification results over different frequency bands. Purple, green, blue, and grey line represent the EC in FOI-N, FOI-1, FOI-2 and FOI-N + FOI-1 + FOI-2, respectively. **(B)** EC with highly discriminative power. The nodes which belong to each RSN in **(B)** are identified by different colors.

We used frequency-specific ECs (all ECs in each FOI) as input features to discriminate patients from controls. As shown in Table 2, our model accurately identified individuals with MDD in each frequency band (accuracy of 84.79%, 75.25%, and 82.20% for FOI-N, FOI-,1 and FOI-2, respectively). Importantly, when combining the EC features across all three frequency bands, we achieved the highest classification accuracy of 89.97%, sensitivity of 92.63%, specificity of 87.32%, and AUC of 0.8831. Figure 5A displays the corresponding average ROC curves for each frequency condition. We further analyzed which EC features possessed high discriminative power. The frequency of each feature selected in all 10-fold cross-validations was calculated to reflect the feature’s contribution to the classification. The top 10 most recognizable EC features in each frequency band are presented in Figure 5B.

**Table 2:** Discriminating the patients with MDD from the HCs by ROC analyses.

FOI	AUC	Accuracy (%)	Sensitivity (%)	Specificity (%)
FOI-N	0.8373	84.79	87.54	81.90
FOI-1	0.7651	75.25	73.61	76.67
FOI-2	0.8163	82.20	86.87	77.34
FOI-N + FOI-1 + FOI-2	0.8831	89.97	92.63	87.32

## 4. Discussion

In this study, we explored the anomalies in EC of triple networks across different frequency bands in MDD by combining the CEEMDAN and mGCA methodologies. Our analysis not only revealed an enhanced mean EC within the CEN in the conventional frequency band, but also a decrease in the mean EC from the SN to the DMN in FOI-1, as well as an increase in the mean EC from the CEN to the SN in FOI-2 in MDD patients. Moreover, a significant association between the mean EC from the CEN to the SN and the HAMD scores was identified in FOI-2 for individuals with MDD. By incorporating EC features across all the three frequency bands, optimal classification performance was achieved. These results reveal frequency-specific alterations in causal influences among triple networks for patients with MDD and highlight the importance of considering multiple frequency bands when developing more precise and dependable biomarkers for disease diagnosis.

Our findings demonstrated that the EC within the triple networks is altered in a frequency-specific manner in patients with MDD. This observation aligns with previous research that has emphasized the importance of examining connectivity patterns across multiple frequency bands in order to fully understand the neurobiological underpinnings of MDD (Wang et al., 2016; Yang et al., 2021). Specifically, we observed a significant increase in the mean EC within the CEN in MDD patients compared to unaffected controls in FOI-N ranging from 0.01 to 0.08 Hz. This finding is consistent with prior studies that have reported altered FC within the CEN in MDD patients (Mulders et al., 2015; Shen et al., 2015; Zhang et al., 2016). The increased EC within the CEN may reflect a compensatory mechanism in response to the disrupted network communications, as the CEN is responsible for higher-order cognitive processes, such as working memory and executive control (Menon, 2011). Alternatively, this alteration might be indicative of a maladaptive change contributing to the cognitive deficits frequently observed in MDD (Mulders et al., 2015; Otte et al., 2016; Kandilarova et al., 2018). Moreover, we found significant decreases in the mean EC from the SN to the DMN in patients compared to controls in FOI-1 (0.12–0.18 Hz). This result aligns with prior findings of disrupted connectivity between the SN and DMN in MDD (Balaev et al., 2018; Fettes et al., 2018; Gong et al., 2019). The SN plays a crucial role in detecting and processing emotionally salient stimuli (Etkin et al., 2011; Seo et al., 2018), while the DMN is implicated in self-referential thinking and rumination (Scheibner et al., 2017). A reduced causal influence from the SN to the DMN might indicate an impaired ability to regulate internal emotional states and a propensity for excessive rumination in MDD patients (Gandelman et al., 2019). In addition, our study demonstrated significant decreases in the mean EC from the CEN to the SN in MDD patients compared to controls in FOI-2 (0.04–0.08 Hz). This finding expands upon previous research that has reported disrupted connectivity between the CEN and SN in MDD (Kaiser et al., 2015). A decreased causal influence from the CEN to the SN might suggest an impaired top-down regulation of emotional processing in MDD patients, potentially contributing to the affective symptom characteristic of the disorder (Kennis et al., 2020). Importantly, our study expands upon existing research by exploring the multi-frequency domain, revealing that EC alterations within the triple networks in MDD may be governed by specific frequency bands. Frequency-specific alterations can reflect distinct biological processes or brain features. BOLD signals at different frequencies may signify differing neuronal activities or interactions. Thus, our finding suggests that varying sensitivities to different frequency bands may exist in the causal interactions among core network structure.

Our findings also revealed that the altered mean EC from CEN to SN in FOI-2 exhibited a significant correlation with HAMD scores in MDD patients, indicating that lower directed interactions correspond to increased disease severity. This observation aligns with prior research demonstrating associations between brain dysconnectivity and depressive symptoms (Kang et al., 2018; Yang et al., 2018; Gandelman et al., 2019). A recent investigation reported correlations between abnormal amygdala connectivity and symptom severity in MDD (Ye et al., 2023), lending further credence to the clinical relevance of our results. Our previous work also indicated that diminished static and dynamic FCs were associated with greater MDD severity (Yao et al., 2019a,b). Importantly, the negative correlations observed exclusively in FOI-2 may represent frequency-specific symptoms of MDD, corroborating earlier findings that Slow-4 (0.027–0.073 Hz) (Yang et al., 2021), overlapping with FOI-2, may hold a crucial role in MDD diagnosis and progression monitoring. These results also suggest that FOI-2 might serve as a specific frequency band reflecting clinical symptoms in MDD patients. Furthermore, our investigation demonstrated that the highest classification accuracy was attained when combining EC features across all three frequency bands (FOI-N, FOI-1, and FOI-2), consistent with other studies reporting enhanced classification accuracy upon considering multiple frequency bands in neuropsychiatric disorders (Chen et al., 2016; Hu et al., 2021). Collectively, these findings underscore the importance of incorporating multiple frequency bands when examining the pathophysiology of MDD and indicate that a comprehensive, multi-frequency approach may yield more precise and reliable biomarkers for the diagnosis and differentiation of patients from controls.

The implications of our findings may extend to two aspects of future MDD therapy. First, this study illuminates the neural pathophysiology underpinning MDD and offers a fresh perspective on frequency-specific dysconnectivity patterns, potentially revealing treatment markers associated with disease severity. The frequency-specific EC alterations identified provide intricate insights into how these functional connections fluctuate across different frequencies. These findings could potentially be harnessed for precise therapeutic interventions, such as neurofeedback or transcranial magnetic stimulation, which can be used to modulate aberrant connectivity patterns in MDD patients (Drysdale et al., 2017). Second, our investigation supplies critical information in the pursuit of clinically valuable diagnostic markers for MDD. Numerous researchers have recently explored the potential of brain connectivity to differentiate MDD patients from unaffected controls (Zhong et al., 2017; Geng et al., 2018; Zhang et al., 2020). Consequently, the identified frequency-specific EC features capable of distinguishing patients from controls with notable accuracy could contribute to the development of more dependable and objective diagnostic instruments, assisting clinicians in the early detection of MDD (Guo et al., 2020). Nevertheless, given the limited sample sizes in this study, the high classification performance warrants validation in future research with larger samples.

There are some limitations that should be noted. First, the resting-state fMRI data acquisition employed a relatively lower repetition time (2 s), constraining the detection of dynamic fluctuations in higher frequency bands (>0.25 Hz). Future research would benefit from utilizing a higher sampling frequency. Second, while Granger causality analysis (GCA) is regarded as an effective method for evaluating EC in resting-state fMRI data, it has been postulated that directional changes might result from hemodynamic coupling differences among distinct brain regions (Pervaiz et al., 2020). Recently, alternative models, specifically the dynamic causal model (DCM)—a hemodynamic model (Friston et al., 2014), have been proposed to detect directed connectivity among hidden neuronal states (Park et al., 2018; Zarghami and Friston, 2020). Consequently, future studies employing DCM to explore frequency-specific reorganizations of EC in MDD patients would be of considerable interest. Third, the patient cohort in this study had prolonged exposure to various antidepressant medications. Prior research has assessed the impact of antidepressants on brain connectivity (Korgaonkar et al., 2019), and it cannot be ruled out that medication effects may have influenced our findings. Nonetheless, previous FC investigations involving high-risk MDD individuals have indicated that altered connectivity between triple networks occurs in the absence of antidepressant treatment (Pawlak et al., 2022). A future study with a never-medicated sample is required to corroborate our findings.

In conclusion, our study revealed frequency-specific alterations in the causal influences among the DMN, SN, and CEN in MDD, with potential ramifications for diagnosis and treatment. These findings enhance our comprehension of the neurobiological underpinnings of MDD and stress the significance of investigating EC patterns within the triple networks across multiple frequency bands. Future research endeavors should build upon these insights to further elucidate the role of frequency-specific EC patterns in MDD pathophysiology, examine their potential as therapeutic targets, and assess their applicability as objective biomarkers for MDD diagnosis.

## Data availability statement

The raw data supporting the conclusions of this article will be made available by the authors, without undue reservation.

## Ethics statement

The studies involving human participants were reviewed and approved by Gansu Provincial Hospital. The patients/participants provided their written informed consent to participate in this study.

## Author contributions

YL designed the study and performed statistical analyses, drafted the manuscript, and approved the final manuscript as submitted. LQ and GL coordinated and carried out the data collection, revised the manuscript, and approved the final manuscript as submitted. ZZ conceptualized the study, critically reviewed the manuscript, and approved the final manuscript as submitted. All authors contributed to the article and approved the submitted version.

## Funding

This work was supported by the National Natural Science Foundation of China (No. 82001918) and by the China Postdoctoral Science Foundation (No. 2020 M681865).

## Conflict of interest

The authors declare that the research was conducted in the absence of any commercial or financial relationships that could be construed as a potential conflict of interest.

The reviewer ZY declared a past co-authorship with the author GL to the handling editor.

## Publisher’s note

All claims expressed in this article are solely those of the authors and do not necessarily represent those of their affiliated organizations, or those of the publisher, the editors and the reviewers. Any product that may be evaluated in this article, or claim that may be made by its manufacturer, is not guaranteed or endorsed by the publisher.

## Footnotes

^1^ http://www.restfmri.net

^2^ https://www.fil.ion.ucl.ac.uk

## References

[ref1] Allen E. A. Damaraju E. Plis S. M. Erhardt E. B. Eichele T. Calhoun V. D. (2014). Tracking whole-brain connectivity dynamics in the resting state. Cereb. Cortex 24, 663–676. doi: 10.1093/cercor/bhs352, PMID: 23146964 PMC3920766

[ref2] Balaev V. Orlov I. Petrushevsky A. Martynova O. (2018). Functional connectivity between salience, default mode and frontoparietal networks in post-stroke depression. J. Affect. Disord. 227, 554–562. doi: 10.1016/j.jad.2017.11.044, PMID: 29169125

[ref3] Bell A. J. Sejnowski T. J. (1995). An information-maximization approach to blind separation and blind deconvolution. Neural Comput. 7, 1129–1159. doi: 10.1162/neco.1995.7.6.1129, PMID: 7584893

[ref4] Buzsáki G. Draguhn A. (2004). Neuronal oscillations in cortical networks. Science 304, 1926–1929. doi: 10.1126/science.1099745 15218136

[ref5] Chen H. Duan X. Liu F. Lu F. Ma X. Zhang Y.. (2016). Multivariate classification of autism spectrum disorder using frequency-specific resting-state functional connectivity—a multi-center study. Prog. Neuro-Psychopharmacol. Biol. Psychiatry 64, 1–9. doi: 10.1016/j.pnpbp.2015.06.014, PMID: 26148789

[ref6] Cheng W. Rolls E. T. Qiu J. Yang D. Ruan H. Wei D.. (2018). Functional connectivity of the precuneus in unmedicated patients with depression. Biol. Psychiatry Cogn. Neurosci. Neuroimaging 3, 1040–1049. doi: 10.1016/j.bpsc.2018.07.008, PMID: 30243643

[ref7] Cole S. R. Voytek B. (2017). Brain oscillations and the importance of waveform shape. Trends Cogn. Sci. 21, 137–149. doi: 10.1016/j.tics.2016.12.008, PMID: 28063662

[ref8] Colominas M. A. Schlotthauer G. Torres M. E. (2014). Improved complete ensemble EMD: a suitable tool for biomedical signal processing. Biomed. Signal Process. Control 14, 19–29. doi: 10.1016/j.bspc.2014.06.009

[ref9] Cortes C. Vapnik V. (1995). Support-vector networks. Mach. Learn. 20, 273–297. doi: 10.1007/BF00994018

[ref10] David O. Guillemain I. Saillet S. Reyt S. Deransart C. Segebarth C.. (2008). Identifying neural drivers with functional MRI: an electrophysiological validation. PLoS Biol. 6, 2683–2697. doi: 10.1371/journal.pbio.0060315, PMID: 19108604 PMC2605917

[ref11] Deshpande G. Hu X. (2012). Investigating effective brain connectivity from fMRI data: past findings and current issues with reference to granger causality analysis. Brain Connect. 2, 235–245. doi: 10.1089/brain.2012.0091, PMID: 23016794 PMC3621319

[ref12] Drysdale A. T. Grosenick L. Downar J. Dunlop K. Mansouri F. Meng Y.. (2017). Resting-state connectivity biomarkers define neurophysiological subtypes of depression. Nat. Med. 23, 28–38. doi: 10.1038/nm.4246, PMID: 27918562 PMC5624035

[ref13] Etkin A. Egner T. Kalisch R. (2011). Emotional processing in anterior cingulate and medial prefrontal cortex. Trends Cogn. Sci. 15, 85–93. doi: 10.1016/j.tics.2010.11.004, PMID: 21167765 PMC3035157

[ref14] Fettes P. W. Moayedi M. Dunlop K. Mansouri F. Vila-Rodriguez F. Giacobbe P.. (2018). Abnormal functional connectivity of Frontopolar subregions in treatment-nonresponsive major depressive disorder. Biol. Psychiatry Cogn. Neurosci. Neuroimaging 3, 337–347. doi: 10.1016/j.bpsc.2017.12.003, PMID: 29628066

[ref15] Fiorenzato E. Strafella A. P. Kim J. Schifano R. Weis L. Antonini A.. (2019). Dynamic functional connectivity changes associated with dementia in Parkinson's disease. Brain 142, 2860–2872. doi: 10.1093/brain/awz192, PMID: 31280293 PMC6736370

[ref16] Friston K. J. Kahan J. Biswal B. Razi A. (2014). A DCM for resting state fMRI. NeuroImage 94, 396–407. doi: 10.1016/j.neuroimage.2013.12.009, PMID: 24345387 PMC4073651

[ref17] Gandelman J. A. Albert K. Boyd B. D. Park J. W. Riddle M. Woodward N. D.. (2019). Intrinsic functional network connectivity is associated with clinical symptoms and cognition in late-life depression. Biol. Psychiatry Cogn. Neurosci. Neuroimaging 4, 160–170. doi: 10.1016/j.bpsc.2018.09.003, PMID: 30392844 PMC6368882

[ref18] Geng X. Xu J. Liu B. Shi Y. (2018). Multivariate classification of major depressive disorder using the effective connectivity and functional connectivity. Front. Neurosci. 12:38. doi: 10.3389/fnins.2018.00038, PMID: 29515348 PMC5825897

[ref19] Gong J. Chen G. Jia Y. Zhong S. Zhao L. Luo X.. (2019). Disrupted functional connectivity within the default mode network and salience network in unmedicated bipolar II disorder. Prog. Neuro-Psychopharmacol. Biol. Psychiatry 88, 11–18. doi: 10.1016/j.pnpbp.2018.06.012, PMID: 29958116

[ref20] Gotlib I. H. Joormann J. (2010). Cognition and depression: current status and future directions. Annu. Rev. Clin. Psychol. 6, 285–312. doi: 10.1146/annurev.clinpsy.121208.131305, PMID: 20192795 PMC2845726

[ref21] Guo M. Wang T. Zhang Z. Chen N. Li Y. Wang Y.. (2020). Diagnosis of major depressive disorder using whole-brain effective connectivity networks derived from resting-state functional MRI. J. Neural Eng. 17:056038. doi: 10.1088/1741-2552/abbc28, PMID: 32987369

[ref22] Hu R. Peng Z. Zhu X. Gan J. Zhu Y. Ma J.. (2021). Multi-band brain network analysis for functional neuroimaging biomarker identification. IEEE Trans. Med. Imaging 40, 3843–3855. doi: 10.1109/TMI.2021.3099641, PMID: 34310294 PMC8931676

[ref23] Kaiser R. H. Andrews-Hanna J. R. Wager T. D. Pizzagalli D. A. (2015). Large-scale network dysfunction in major depressive disorder: a meta-analysis of resting-state functional connectivity. JAMA Psychiat. 72, 603–611. doi: 10.1001/jamapsychiatry.2015.0071, PMID: 25785575 PMC4456260

[ref24] Kandilarova S. Stoyanov D. Kostianev S. Specht K. (2018). Altered resting state effective connectivity of anterior insula in depression. Front. Psych. 9:83. doi: 10.3389/fpsyt.2018.00083, PMID: 29599728 PMC5862800

[ref25] Kang L. Zhang A. Sun N. Liu P. Yang C. Li G.. (2018). Functional connectivity between the thalamus and the primary somatosensory cortex in major depressive disorder: a resting-state fMRI study. BMC Psychiatry 18:339. doi: 10.1186/s12888-018-1913-6, PMID: 30340472 PMC6194586

[ref26] Kennis M. Gerritsen L. van Dalen M. Williams A. Cuijpers P. Bockting C. (2020). Prospective biomarkers of major depressive disorder: a systematic review and meta-analysis. Mol. Psychiatry 25, 321–338. doi: 10.1038/s41380-019-0585-z, PMID: 31745238 PMC6974432

[ref27] Kim J. Criaud M. Cho S. S. Mihaescu A. Coakeley S. Ghadery C.. (2017). Abnormal intrinsic brain functional network dynamics in Parkinson's disease. Brain 140, 2955–2967. doi: 10.1093/brain/awx233, PMID: 29053835 PMC5841202

[ref28] Korgaonkar M. S. Goldstein-Piekarski A. N. Fornito A. Williams L. M. (2019). Intrinsic connectomes are a predictive biomarker of remission in major depressive disorder. Mol. Psychiatry 25, 1537–1549. doi: 10.1038/s41380-019-0574-2 31695168 PMC7303006

[ref29] Li G. Rossbach K. Zhang A. Liu P. Zhang K. (2018). Resting-state functional changes in the precuneus within first-episode drug-naive patients with MDD. Neuropsychiatr. Dis. Treat. 14, 1991–1998. doi: 10.2147/NDT.S168060, PMID: 30122932 PMC6086096

[ref30] Liao W. Ding J. Marinazzo D. Xu Q. Wang Z. Yuan C.. (2011). Small-world directed networks in the human brain: multivariate granger causality analysis of resting-state fMRI. NeuroImage 54, 2683–2694. doi: 10.1016/j.neuroimage.2010.11.007, PMID: 21073960

[ref31] Martino M. Magioncalda P. Huang Z. Conio B. Piaggio N. Duncan N. W.. (2016). Contrasting variability patterns in the default mode and sensorimotor networks balance in bipolar depression and mania. Proc. Natl. Acad. Sci. U. S. A. 113, 4824–4829. doi: 10.1073/pnas.1517558113, PMID: 27071087 PMC4855585

[ref32] Mathers C. D. Loncar D. (2006). Projections of global mortality and burden of disease from 2002 to 2030. PLoS Med. 3:e442. doi: 10.1371/journal.pmed.0030442, PMID: 17132052 PMC1664601

[ref33] Menon V. (2011). Large-scale brain networks and psychopathology: a unifying triple network model. Trends Cogn. Sci. 15, 483–506. doi: 10.1016/j.tics.2011.08.003 21908230

[ref34] Mulders P. C. van Eijndhoven P. F. Schene A. H. Beckmann C. F. Tendolkar I. (2015). Resting-state functional connectivity in major depressive disorder: a review. Neurosci. Biobehav. Rev. 56, 330–344. doi: 10.1016/j.neubiorev.2015.07.014 26234819

[ref35] Otte C. Gold S. M. Penninx B. W. Pariante C. M. Etkin A. Fava M.. (2016). Major depressive disorder. Nat. Rev. Dis. Primers. 2, 1–20. doi: 10.1038/nrdp.2016.65 27629598

[ref36] Park H. J. Friston K. J. Pae C. Park B. Razi A. (2018). Dynamic effective connectivity in resting state fMRI. NeuroImage 180, 594–608. doi: 10.1016/j.neuroimage.2017.11.033 29158202 PMC6138953

[ref37] Pawlak M. Bray S. Kopala-Sibley D. C. (2022). Resting state functional connectivity as a marker of internalizing disorder onset in high-risk youth. Sci. Rep. 12:21337. doi: 10.1038/s41598-022-25805-y, PMID: 36494495 PMC9734132

[ref38] Peng X. Lau W. K. W. Wang C. Ning L. Zhang R. (2020). Impaired left amygdala resting state functional connectivity in subthreshold depression individuals. Sci. Rep. 10:17207. doi: 10.1038/s41598-020-74166-x, PMID: 33057046 PMC7560839

[ref39] Pereira F. Mitchell T. Botvinick M. (2009). Machine learning classifiers and fMRI: a tutorial overview. NeuroImage 45, S199–S209. doi: 10.1016/j.neuroimage.2008.11.007, PMID: 19070668 PMC2892746

[ref40] Pervaiz U. Vidaurre D. Woolrich M. W. Smith S. M. (2020). Optimising network modelling methods for fMRI. NeuroImage 211:116604. doi: 10.1016/j.neuroimage.2020.116604, PMID: 32062083 PMC7086233

[ref41] Rolls E. T. Cheng W. Gilson M. Qiu J. Hu Z. Ruan H.. (2018). Effective connectivity in depression. Biol. Psychiatry Cogn. Neurosci. Neuroimaging 3, 187–197. doi: 10.1016/j.bpsc.2017.10.004, PMID: 29529414

[ref42] Samaha J. Iemi L. Haegens S. Busch N. A. (2020). Spontaneous brain oscillations and perceptual decision-making. Trends Cogn. Sci. 24, 639–653. doi: 10.1016/j.tics.2020.05.004, PMID: 32513573

[ref43] Scheibner H. J. Bogler C. Gleich T. Haynes J. D. Bermpohl F. (2017). Internal and external attention and the default mode network. NeuroImage 148, 381–389. doi: 10.1016/j.neuroimage.2017.01.044 28110087

[ref44] Seo R. Stocco A. Prat C. S. (2018). The bilingual language network: differential involvement of anterior cingulate, basal ganglia and prefrontal cortex in preparation, monitoring, and execution. NeuroImage 174, 44–56. doi: 10.1016/j.neuroimage.2018.02.010, PMID: 29486320

[ref45] Shen T. Li C. Wang B. Yang W. M. Zhang C. Wu Z.. (2015). Increased cognition connectivity network in major depression disorder: a FMRI study. Psychiatry Investig. 12, 227–234. doi: 10.4306/pi.2015.12.2.227, PMID: 25866524 PMC4390594

[ref46] Tu Y. Fu Z. Zeng F. Maleki N. Lan L. Li Z.. (2019). Abnormal thalamocortical network dynamics in migraine. Neurology 92, e2706–e2716. doi: 10.1212/WNL.0000000000007607, PMID: 31076535 PMC6556096

[ref47] Wang L. Kong Q. Li K. Su Y. Zeng Y. Zhang Q.. (2016). Frequency-dependent changes in amplitude of low-frequency oscillations in depression: a resting-state fMRI study. Neurosci. Lett. 614, 105–111. doi: 10.1016/j.neulet.2016.01.012, PMID: 26797652

[ref48] Yan C. G. Chen X. Li L. Castellanos F. X. Bai T. J. Bo Q. J.. (2019). Reduced default mode network functional connectivity in patients with recurrent major depressive disorder. Proc. Natl. Acad. Sci. U. S. A. 116, 9078–9083. doi: 10.1073/pnas.1900390116, PMID: 30979801 PMC6500168

[ref49] Yang H. Chen X. Chen Z. B. Li L. Li X. Y. Castellanos F. X.. (2021). Disrupted intrinsic functional brain topology in patients with major depressive disorder. Mol. Psychiatry 26, 7363–7371. doi: 10.1038/s41380-021-01247-2, PMID: 34385597 PMC8873016

[ref50] Yang Y. Cui Q. Pang Y. Chen Y. Tang Q. Guo X.. (2021). Frequency-specific alteration of functional connectivity density in bipolar disorder depression. Prog. Neuro-Psychopharmacol. Biol. Psychiatry 104:110026. doi: 10.1016/j.pnpbp.2020.110026 32621959

[ref51] Yang Z. Gu S. Honnorat N. Linn K. A. Shinohara R. T. Aselcioglu I.. (2018). Network changes associated with transdiagnostic depressive symptom improvement following cognitive behavioral therapy in MDD and PTSD. Mol. Psychiatry 23, 2314–2323. doi: 10.1038/s41380-018-0201-7, PMID: 30104727 PMC13112374

[ref52] Yao Z. Shi J. Zhang Z. Zheng W. Hu T. Li Y.. (2019a). Altered dynamic functional connectivity in weakly-connected state in major depressive disorder. Clin. Neurophysiol. 130, 2096–2104. doi: 10.1016/j.clinph.2019.08.009, PMID: 31541987

[ref53] Yao Z. Zou Y. Zheng W. Zhang Z. Li Y. Yu Y.. (2019b). Structural alterations of the brain preceded functional alterations in major depressive disorder patients: evidence from multimodal connectivity. J. Affect. Disord. 253, 107–117. doi: 10.1016/j.jad.2019.04.064, PMID: 31035211

[ref54] Ye Y. Wang C. Lan X. Li W. Fu L. Zhang F.. (2023). Abnormal amygdala functional connectivity in MDD patients with insomnia complaints. Psychiatry Res. Neuroimaging 328:111578. doi: 10.1016/j.pscychresns.2022.111578, PMID: 36525761

[ref55] Zarghami T. S. Friston K. J. (2020). Dynamic effective connectivity. NeuroImage 207:116453. doi: 10.1016/j.neuroimage.2019.116453 31821868

[ref56] Zhang B. Li M. Qin W. Demenescu L. R. Metzger C. D. Bogerts B.. (2016). Altered functional connectivity density in major depressive disorder at rest. Eur. Arch. Psychiatry Clin. Neurosci. 266, 239–248. doi: 10.1007/s00406-015-0614-0, PMID: 26265034

[ref57] Zhang Z. Liu G. Yao Z. Zheng W. Xie Y. Hu T.. (2018). Changes in dynamics within and between resting-state subnetworks in juvenile myoclonic epilepsy occur at multiple frequency bands. Front. Neurol. 9:448. doi: 10.3389/fneur.2018.00448, PMID: 29963004 PMC6010515

[ref58] Zhang Y. Wu W. Toll R. T. Naparstek S. Maron-Katz A. Watts M.. (2020). Identification of psychiatric disorder subtypes from functional connectivity patterns in resting-state electroencephalography. Nat. Biomed. Eng. 5, 309–323. doi: 10.1038/s41551-020-00614-8 PMC8053667 33077939

[ref59] Zhong X. Shi H. Ming Q. Dong D. Zhang X. Zeng L. L.. (2017). Whole-brain resting-state functional connectivity identified major depressive disorder: a multivariate pattern analysis in two independent samples. J. Affect. Disord. 218, 346–352. doi: 10.1016/j.jad.2017.04.040, PMID: 28499208

[ref60] Zuo X. N. Di Martino A. Kelly C. Shehzad Z. E. Gee D. G. Klein D. F.. (2010). The oscillating brain: complex and reliable. NeuroImage 49, 1432–1445. doi: 10.1016/j.neuroimage.2009.09.037, PMID: 19782143 PMC2856476

