# Supplementary material for: Frequency specificity of aberrant triple networks in major depressive disorder: a resting-state effective connectivity study
Source: Front Neurosci. 2023 Jun 30;17:1200029. doi: 10.3389/fnins.2023.1200029 (PMC10347531; doi:10.3389/fnins.2023.1200029)
Supplement: Supplementary file 1 [file Table_1.DOCX]

Supplementary Material

Frequency specificity of aberrant triple networks in major depressive disorder: a resting-state effective connectivity study

Ying Li, Linze Qian, Gang Li*, Zhe Zhang*

*** Correspondence:** Gang Li: [ligang@zjnu.cn](mailto:ligang@zjnu.cn) or Zhe Zhang: zhangz@hznu.edu.cn

# Supplementary Tables

**Supplementary Table1.** Peak activation information of the 21 ICs

| **ICs** | **Anatomical regions** | **AAL** | **BA** | **MNI coordinates^*^** | | | **Cluster size** | ***T*-value** |
| --- | --- | --- | --- | --- | --- | --- | --- | --- |
|  |  |  |  | X | Y | Z |  |  |
| **DMN (9)** |  |  |  |  |  |  |  |  |
| ■ IC21 | B medial frontal gyrus | Frontal_Sup_Medial_L & _R | 11 | -9 | 33 | -18 | 904 | 40.46 |
| ■ IC33 | B anterior cingulate cortex | Cingulum_Ant_L & _R | 32 | -3 | 39 | 6 | 911 | 47.38 |
| ■ IC52 | B precuneus | Precuneus_L & _R | 7 | 3 | -54 | 48 | 915 | 39.42 |
| ■ IC54 | B posterior cingulate cortex | Cingulum_Post_L & _R | 30 | -12 | -57 | 9 | 941 | 43.91 |
| ■ IC59 | B precuneus | Precuneus_L & _R | 7 | -9 | -69 | 30 | 782 | 24.42 |
| ■ IC78 | L inferior parietal lobule | Parietal_Inf_L | 40 | -45 | -36 | 42 | 421 | 36.41 |
|  | R inferior parietal lobule | Parietal_Inf_R | 40 | 48 | -30 | 42 | 372 | 34.56 |
| ■ IC86 | R inferior parietal lobule | Parietal_Inf_R | 40 | 33 | -66 | 39 | 853 | 35.80 |
| ■ IC92 | L inferior parietal lobule | Parietal_Inf_L | 40 | -48 | -54 | 42 | 501 | 46.60 |
|  | R inferior parietal lobule | Parietal_Inf _R | 40 | 54 | -48 | 42 | 176 | 28.81 |
| ■ IC100 | B superior frontal gyrus | Frontal_Sup_L &_R | 8 | -12 | 21 | 60 | 627 | 23.21 |
| **SN (4)** |  |  |  |  |  |  |  |  |
| ■ IC32 | L anterior insula | Insula_L | 47 | -33 | 21 | -6 | 360 | 43.11 |
|  | R anterior insula | Insula_R | 47 | 30 | 24 | -6 | 396 | 42.93 |
| ■ IC65 | L temporal pole | Temporal_Pole_Mid_L | 38 | -42 | 12 | -33 | 440 | 28.96 |
|  | R temporal pole | Temporal_Pole_Mid_R | 38 | 45 | 18 | -33 | 510 | 26.76 |
| ■ IC69 | R posterior insula | Insula_R | 13 | 30 | 3 | 3 | 664 | 24.63 |
| ■ IC71 | L posterior insula | Insula_L | 13 | -42 | 3 | 3 | 742 | 29.36 |
| **CEN (8)** |  |  |  |  |  |  |  |  |
| ■ IC53 | B middle cingulate cortex | Cingulum_Mid_L & _R | 23 | -3 | -30 | 24 | 723 | 50.70 |
| ■ IC61 | B middle cingulate cortex | Cingulum_Mid_L & _R | 24 | -6 | 0 | 36 | 801 | 33.50 |
| ■ IC73 | L middle frontal gyrus | Frontal_Mid_L | 9 | -3 | 45 | 33 | 928 | 41.13 |
| ■ IC75 | L middle frontal gyrus | Frontal_Mid_L | 10 | -27 | 48 | 21 | 513 | 34.44 |
|  | R middle frontal gyrus | Frontal_Mid_R | 10 | 30 | 51 | 21 | 398 | 32.50 |
| ■ IC77 | L inferior frontal gyrus | Frontal_Inf_Tri_L | 47 | -54 | 21 | 3 | 561 | 42.58 |
| ■ IC87 | R inferior frontal gyrus | Frontal_Inf_Tri_R | 46 | 51 | 24 | 12 | 926 | 33.44 |
| ■ IC88 | R inferior frontal gyrus | Frontal_Inf_Tri_R | 46 | -39 | 42 | -3 | 515 | 34.24 |
| ■ IC96 | L inferior frontal gyrus | Frontal_Inf_Tri_L | 45 | -60 | 21 | 6 | 331 | 21.59 |
|  | R inferior frontal gyrus | Frontal_Inf_Tri_R | 45 | 60 | 18 | 18 | 318 | 20.87 |

^*^The coordinates are peak voxel coordinates of the one-sample t-test results for each component spatial maps of all participants. A color-coded legend of each IC number matches to the overlaid colors of the spatial maps in Figure 2. Abbreviations: ICs, independent components; BA, Brodmann area; L, left; R, right; B, bilateral.
